# Optimizing an Osteosarcoma-Fibroblast Coculture Model to Study Antitumoral Activity of Magnesium-Based Biomaterials

**DOI:** 10.3390/ijms21145099

**Published:** 2020-07-19

**Authors:** Philipp Globig, Regine Willumeit-Römer, Fernanda Martini, Elisa Mazzoni, Bérengère J.C. Luthringer-Feyerabend

**Affiliations:** 1 Institute of Materials Research, Division for Metallic Biomaterials, Helmholtz-Zentrum Geesthacht (HZG), 21502 Geesthacht, Germany; philipp.globig@hzg.de (P.G.); regine.willumeit@hzg.de (R.W.-R.); 2 Department of Medical Sciences, University of Ferrara, 44121 Ferrara, Italy; fernanda.martini@unife.it (F.M.); elisa.mazzoni@unife.it (E.M.)

**Keywords:** cancer, osteosarcoma, magnesium, coculture, degradation

## Abstract

Osteosarcoma is among the most common cancers in young patients and is responsible for one-tenth of all cancer-related deaths in children. Surgery often leads to bone defects in excised tissue, while residual cancer cells may remain. Degradable magnesium alloys get increasing attention as orthopedic implants, and some studies have reported potential antitumor activity. However, most of the studies do not take the complex interaction between malignant cells and their surrounding stroma into account. Here, we applied a coculture model consisting of green fluorescent osteosarcoma cells and red fluorescent fibroblasts on extruded Mg and Mg–6Ag with a tailored degradation rate. In contrast to non-degrading Ti-based material, both Mg-based materials reduced relative tumor cell numbers. Comparing the influence of the material on a sparse and dense coculture, relative cell numbers were found to be statistically different, thus relevant, while magnesium alloy degradations were observed as cell density-independent. We concluded that the sparse coculture model is a suitable mechanistic system to further study the antitumor effects of Mg-based material.

## 1. Introduction

Osteosarcoma (OS) is the most common malignancy of primary bone arising from mesenchymal (bone-forming) cells. This tumor is predominantly found in children and adolescents, with 75% of patients ranging from 15 to 25 years [1]. OS resides in the eighth position among the most common cancers in children (around 2.4%) and accounts for around 8.6% (bones and joints) of cancer-related deaths in children [2,3,4]. The prevalence of osteosarcoma in young patients is increasing to 6–8 million/year [5,6,7,8]. The World Health Organization classifies OS into central (or medullary; 90% of reported cases) and surface (peripheral) tumors, both with various subtypes [1]. Low-grade osteosarcomas can be treated by surgical resection, which can lead to complications during treatment, including bone defects from excised tumor tissue [1,9]. High-grade diagnosed tumors require neoadjuvant and adjuvant chemotherapies, e.g., applying methotrexate, doxorubicin, and cisplatin [9,10,11]. Long-lasting systemic treatments, such as chemotherapy, often come along with severe side effects like nausea and vomiting that dramatically deteriorate the everyday life of the patients. Promising alternatives to overcome these side effects are drug delivery systems, for instance, liposomes and polymer micelles for drug encapsulation, which directly target the tumor side [12].

Another novel approach combining temporary bone replacement for small bone defects after tumor resection and local treatment could be the use of magnesium (Mg)-based biomaterials. Owing to their good biocompatibility and full degradability in physiological environments [13], Mg materials have already been widely discussed for orthopedic applications. Mg degradation is accompanied by a constant ion and hydrogen gas (H_2_) release, as well as increasing pH and osmolality at the material surface microenvironment. These surface-near effects may also specifically affect residual tumor cells that are inaccessible for surgery if the degradation is tailored for exactly this application. Some recent studies have already reported a certain cytotoxic effect of Mg degradation on tumor cells [14,15,16]. Furthermore, alloying of Mg with elements like silver (Ag) holds the potential to additionally influence the degradation rate, mechanical properties, and biological response. Mg–Ag alloys have been already shown to have antibacterial effects [17], and they may also prevent carcinogenic progression as Ag also possesses anticancer properties [18,19]. Ensuring tumor cell-specific cytotoxicity and the simultaneous integrity of adjacent healthy cells is one major challenge when investigating Mg-based materials for the use in cancer therapy. Therefore, tailoring the degradation rate for this purpose is important. While Mg materials in orthopedic applications require very low degradation rates to avoid damages in the adjacent tissues and increase the implant lifetime, tumor therapy approaches demand other strategies. The degradation rate and resulting surface-near effects (increased pH, osmolality, and H_2_ release) should be tailored high enough to kill tumor cells but as low as possible to ensure the integrity of the surrounding healthy tissue. Additionally, such a tumor therapy may benefit from a higher tumor cell sensitivity towards Mg degradation compared to healthy cells, as described by others [20].

Furthermore, it is important to consider the complexity of the tumor microenvironment (TME). Tumors do not only consist of malignant tumor cells but also recruit different cell types into their proximity, where non-tumorous cells often have a positive effect on tumor growth and progression [21,22]. These cells include fibroblasts and cells of the vasculature and immune system summarized in the stroma. Fibroblasts play a very specific role in the TME since they are responsible for the communication of tumor and stromal cells by secreting cytokines [23] and seem to regulate progression and metastases of tumors [24,25]. Multiple studies show that Mg may intervene in this communication between tumor cells and stroma [26,27,28,29]. To enable an in-depth study of the Mg influence on the interaction of tumor and stroma, a meaningful cell model is required. Coculture models should be preferred over simple monoculture systems since they allow the interaction of cell types (transmission of cytokines) and depict in-vivo situations better. Furthermore, a direct coculture allows the proximity and physical interaction of the different cell types. Coculture models, including tissue-specific tumor cells and fibroblasts, are generally accepted as tumor models not only for OS [30] but also for other tumor types, such as mammary cancer [31,32] or pancreatic tumor [33,34].

Based on these publications, we established a 1:1 coculture system consisting of green fluorescent osteosarcoma cells (Saos-eGFP) and red fluorescent dermal fibroblasts (RF fibroblasts) in direct contact with the Mg material. This chosen ratio was in accordance with in vivo reported carcinoma-percentage derived from the carcinoma-stromal ratio, i.e., 20% to 90% (here 50%) [35]. In contrast to the aforementioned studies that used a coculture test system on inert tissue culture plastic, we were challenged with the degradation of Mg-based materials, that is, on the one hand, influenced by the cells and, on the other hand, can impact the cells [36,37,38,39,40,41]. The aim of this study was to investigate the influence of Mg-based materials on tumor and healthy cells in future studies. To identify influences of our material on our coculture and find the optimal seeding conditions, two cell densities, namely, a sparse (10,000 cells in total) and a dense (50,000 cells in total) coculture, were compared regarding their suitability for an appropriate coculture model. Thus, we investigated whether Mg and Mg–6Ag have a significant influence on the ratio of both seeded cell types. Furthermore, we explored possible cell-dependent influences on the material degradation, namely, the mean degradation rate (MDR), pH, osmolality, and ion release, to exclude that Mg degradation effects cause differences between the sparse and dense model.

## 2. Results

### 2.1. Effect of Extruded Mg and Mg–6Ag on Tumor Cell to Healthy Cell Number Ratio

**Figure 1:**
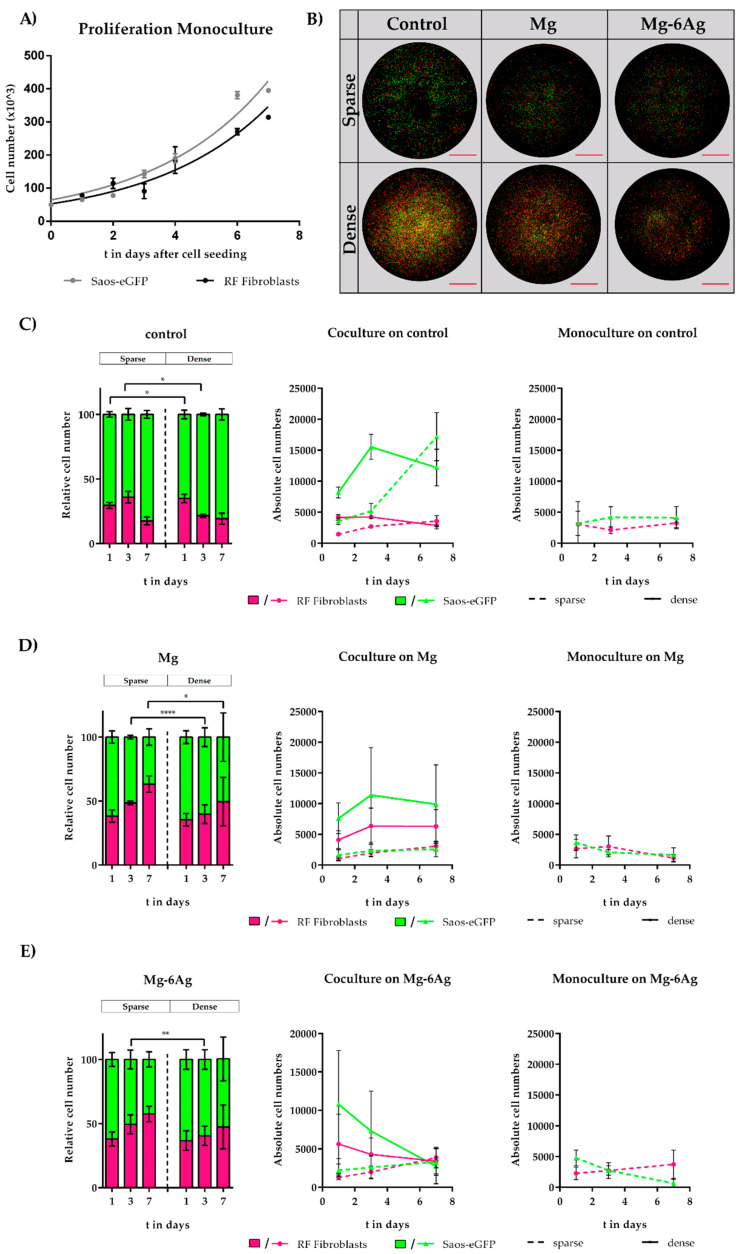
Influence of Mg-based materials on relative healthy and tumor cell numbers. (**A**) Cell proliferation of monocultures on tissue culture plastic. Significance differences between proliferation rates of Saos-eGFP (green fluorescent osteosarcoma cells) and RF fibroblasts (red fluorescent dermal fibroblasts) were obtained via extra sum-of-squares F test of non-linear regression curves. (**B**) Exemplary and representative pictures of the sparse and dense coculture on control (Ti–6Al–4V), Mg, and Mg–6Ag at day 1. Scale bar is 2.5 mm. (**C–E**) Evolution of cell populations (i.e., relative cell numbers, absolute cell numbers) of the tumor (Saos-eGFP; green) and healthy cells (RF fibroblasts; purple) in coculture and monoculture over seven days. Relative and absolute cell numbers were presented as the arithmetical mean ± SD of three independent experiments. Significance differences between samples of the respective time points from the dense and sparse model were obtained via a Mann–Whitney test (*n* = 9); * = *p* < 0.05; ** = *p* < 0.01; **** = *p* < 0.0001.

**Figure 2:**
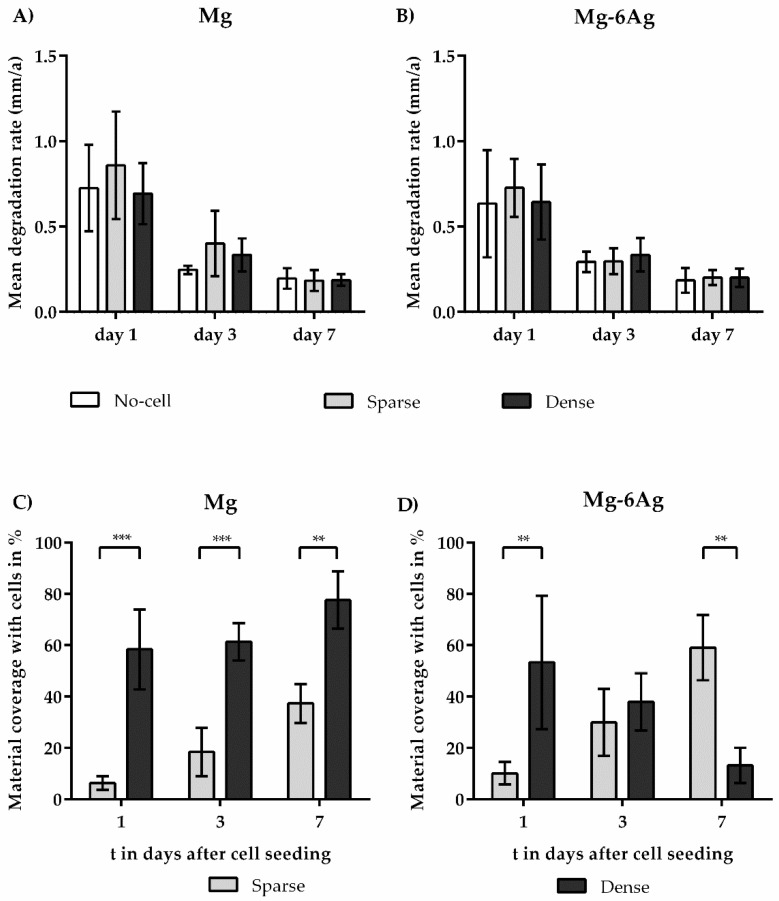
Comparison of mean degradation rates (MDRs) and cell densities on extruded Mg and Mg–6Ag. (**A**,**B**) MDR and (**C**,**D**) respective proportions of material coverage were presented as the arithmetical mean ± SD of three independent experiments. Significance differences between samples of the respective time points from no-cell control, the dense, and sparse model were obtained via a Kruskal–Wallis H test with Dunn’s multiple comparison test (**A**,**B**) or via a Mann–Whitney test (**C**,**D**) (*n* = 9); ** = *p* < 0.01, *** = *p* < 0.001.

**Figure A1:**
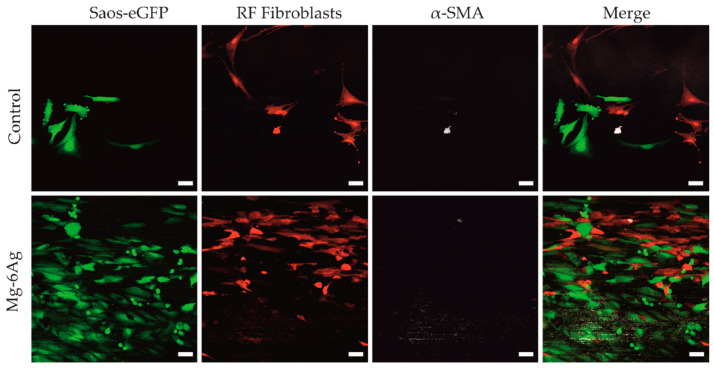
Exclusion of cancer-associated fibroblast (CAF) phenotype for RF fibroblasts. Saos-eGFP (green) and RF fibroblasts (red) were stained for α-smooth muscle actin (α-SMA) (white). RF fibroblasts were negative for α-SMA, indicating that they did not transform to CAFs in the presence of Saos-eGFP. Scale bar is 10 µm.

First, both cell proliferation rates were confirmed to be similar over a culture period of seven days (Figure 1A), ascertaining that further obtained observations were not biased. It was further confirmed that fibroblasts did not acquire a cancer-associated fibroblastic phenotype in coculture with Saos-eGFP via α-smooth muscle actin (α-SMA) staining (Appendix A Figure A1). Then, the difference of relative cell numbers between the sparse (10,000 cells in total) and dense (50,000 cells in total) coculture model (tumor cell:healthy cell ratio 1:1) on Mg and Mg–6Ag degradation was tested. The materials were chosen to compare the effects of degradation products (Mg samples, degradation products: H-release, pH change, increase of Mg ions in the vicinity of the material) with the toxicity, which should come from the functionalization of the material by Ag (Mg–6Ag samples). The degradation rate was similar for both materials (see Figure 2 for details) and in a range that is usually well tolerated by cells. Microscopic images of the whole surface of the disk (Figure 1B) were taken one, three, and seven days after seeding. Cells were counted, and the resulting ratios were determined (Figure 1C–E). Comparing the different materials, it seemed that the degrading Mg and Mg–6Ag favored the growth of healthy fibroblasts compared to the control (Ti–6Al–4V). Within the period of seven days, the cell ratio of tumor cells (Saos-eGFP) declined on Mg from 65 to 50% in the dense model and from 62 to 37% in the sparse model. The healthy cell ratio (red fluorescent or RF fibroblasts) increased on Mg from 35 to 50% in the dense model and 38 to 63% in the sparse model. Seeding on Mg–6Ag showed similar results (tumor cells: 68 to 50%, 62 to 42%; healthy cells: 32 to 50%, 38 to 58%). Opposite results were obtained with non-degrading Ti–6Al–4V (control). Here, the ratio of tumor cells increased from 65 to 81% (dense) and 71 to 83% (sparse model), while the relative cell number of healthy cells decreased from 35 to 19% (dense) and 30 to 18% (sparse model) within seven days. Interestingly, the proportion of fibroblasts was significantly higher in the sparse model compared to the dense coculture model on day 3. Furthermore, on extruded Mg and Mg–6Ag, the proportion of fibroblasts tended to be higher in the sparse model compared to the dense coculture model. Absolute cell numbers of Saos-eGFP and RF fibroblasts did not show significant changes between coculture and monocultures. Though for the coculture in control, absolute tumor cell numbers (sparse model) strongly increased compared to the tumor cells in monoculture within seven days. Regarding Mg and Mg–6Ag, tumor cell numbers were decreasing in the monocultures while remaining constant or slightly increased in the coculture.

### 2.2. Comparison of Material Degradation Rates, pH, and Osmolalities

The viability of cells on cytocompatible Mg-based materials was majorly influenced by material degradation, namely, the mean degradation rate (MDR) accompanied by, e.g., a certain increase in pH and osmolality. The MDR was determined via mass loss at days 1, 3, and 7 after cell seeding. Figure 2 shows the comparison of MDR and material coverage for Mg and Mg–6Ag. MDR of both Mg and Mg–6Ag did not differ significantly between the dense and sparse coculture models. Furthermore, there was no significant difference for MDR between cell-seeded and no-cell samples (Figure 2A,B). However, the proportion of material surface that was covered by cells differed significantly between the sparse and dense coculture model (except for Mg–6Ag on day 3) (Figure 2C,D). On Mg, cell density elevated from 58 to 78% in the dense model and from 6 to 37% in the sparse coculture model within seven days. On Mg–6Ag, the sparse model coverage rose from 10 to 61%, whereas in the dense model, it diminished from 59 to 13%. 

**Figure 3:**
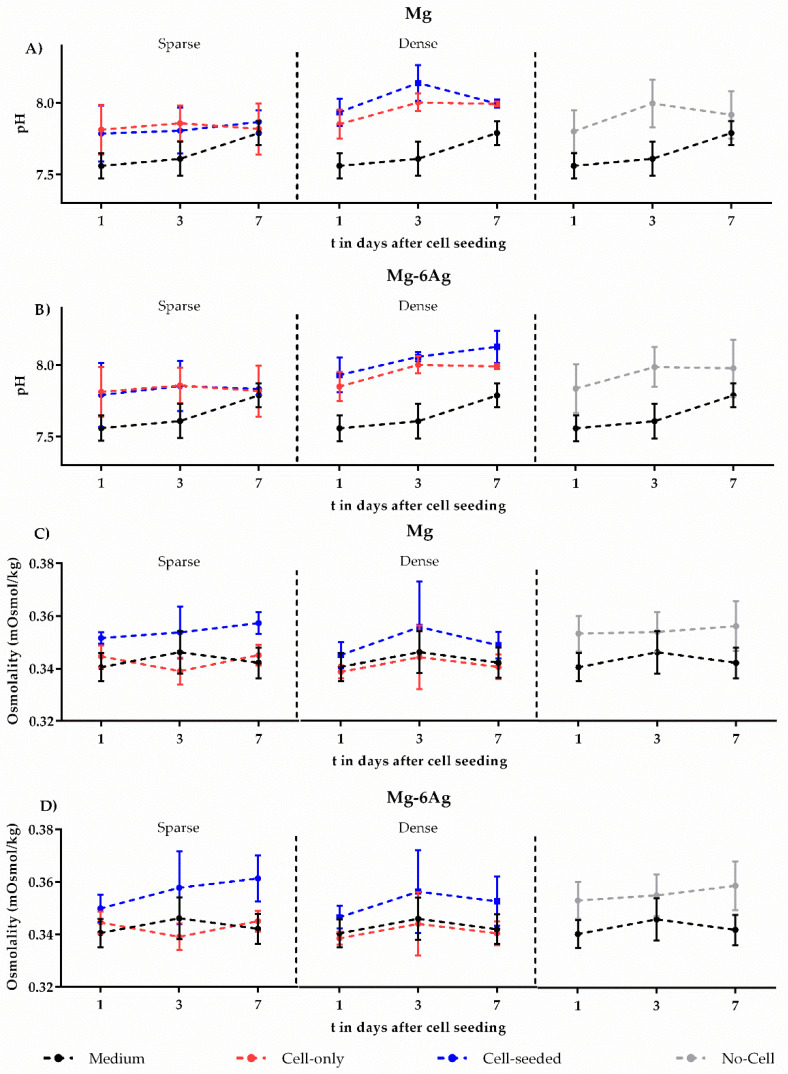
Measurement of pH and osmolality. (**A**,**B**) pH and (**C**,**D**) osmolality of cell-seeded (sparse/dense) and no-cell control for up to seven days. Osmolality and pH values were presented as the arithmetical mean ± SD of three independent experiments. Significance differences between samples of the respective time points from no-cell control, the dense, and sparse model were obtained via a Kruskal–Wallis H test with Dunn’s multiple comparison test (*n* = 9).

Furthermore, the pH and osmolalities were measured one, three, and seven days after cell seeding. Figure 3 shows the pH and osmolality for cell-seeded samples (sparse/dense) and no-cell controls for up to seven days. There was no significant change in pH and osmolality for both coculture models.

### 2.3. Surface Topology of Initial and Degraded Mg and Mg–6Ag

**Figure 4:**
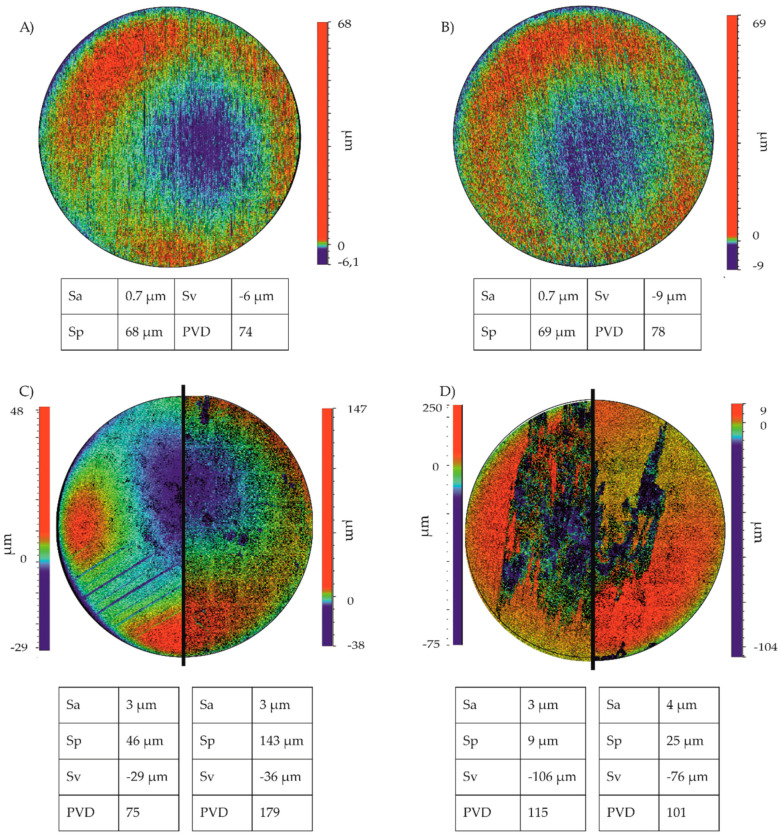
The surface topology of Mg and Mg–6Ag. (**A**) Mg and (**B**) Mg–6Ag as samples in an initial state after grinding are shown. (**C**) The surface topology of Mg and (**D**) Mg–6Ag seeded with the sparse (right half) and dense coculture (left half) after degradation and removal of the degradation layer. To compare the surface morphologies of respective samples with the sparse and dense coculture, the average roughness (Sa), the maximum peak height (Sp), the maximum valley depth (Sv), and the peak-valley difference (PVD) are shown.

To investigate possible influences of the material surface on the proliferation of the cells, images of the surface topology were taken using a white light interferometer (Figure 4). Color scale bars indicated the range between the highest point (peak) and the lowest point (valley) on the material surface. Images of Mg and Mg–6Ag in an initial state after grinding are shown in Figure 4A,B. The investigated parameters, namely, average roughness (Sa), the maximum peak height (Sp), the maximum valley depth (Sv), and the peak-valley difference (PVD), were comparable for Mg and Mg–6Ag. Furthermore, the surface morphologies of the sparse (right half) and dense (left half) coculture after seven days degradation and after removal of the degradation layer are shown for Mg (Figure 4C) and Mg–6Ag (Figure 4D). On both Mg and Mg–6Ag, the average roughness did not differ but was increased compared to the samples in the initial state. On Mg, the PVD of the sample with the sparse model was increased compared to the sample with the dense coculture. In contrast to that, the PVD of both Mg–6Ag samples was comparable.

### 2.4. Quantification of Alloying Elements in the Supernatant

**Figure 5:**
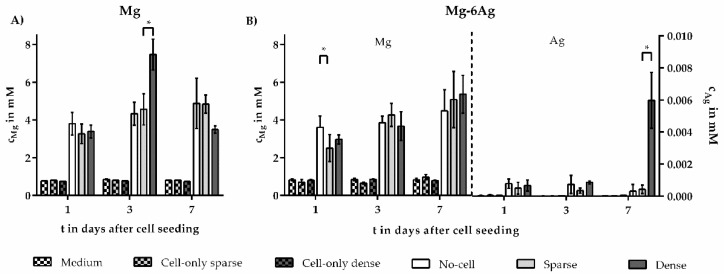
Quantification of Mg and Ag contents in degradation supernatants. (**A**) Mg content quantified using atomic absorption spectroscopy (AAS). (**B)** Co-existing Mg and Ag content in the supernatant of degrading Mg–6Ag quantified by inductively-coupled plasma-mass spectrometry (ICP-MS). Mg and Ag contents of cell-seeded samples were corrected by subtracting cell-only control; no-cell control corrected by subtracting medium. Mg and Ag contents were presented as the arithmetical mean ± SD of three independent experiments. Significance differences between samples of the respective time points from no-cell control, the dense, and sparse model were obtained via a Kruskal–Wallis H test with Dunn’s multiple comparison test (*n* = 6 or 9); * = *p* < 0.05.

To investigate possible anti-cancerous effects of alloying elements, ion releases were quantified by atomic absorption spectroscopy (AAS) or inductively-coupled plasma-mass spectrometry (ICP-MS) in the supernatant after 1, 3, and 7 days culture for the sparse and dense coculture models (Figure 5).

Mg concentration increased over time, increasing from 2.5–5.0 mM in the sparse model and from 2.2–4.6 mM in the dense model. Comparing the ion release after Mg–6Ag degradation, Mg and Ag ion concentrations in the sparse and dense coculture model did not differ significantly. This was also true when comparing respective cell-seeded and cell-free samples, except for control and sparse model at day one. In the sparse model, Ag ion concentration scattered around 0.4 nM, while in the dense coculture model, it rose from 0.6 to 0.8 nM during the first three days and elevated to 6 nM until day seven. Mg ion release after Mg degradation showed majorly no significant difference between the sparse and dense coculture model, except at day three.

## 3. Discussion

In our study, we used a 1:1 coculture (starting condition) of green fluorescent human osteosarcoma cells (Saos-eGFP) and red fluorescent human dermal fibroblasts to establish a simplified in vitro osteosarcoma model. This mechanistic model should enhance the relevance of prospective studies that will test the influence of Mg-based biomaterials and degradation-induced surface effects on tumor and healthy cells of the TME. The coculture model established in our study created a more complex environment, which was closer to the actual in vivo situation compared to using a monoculture of tumor cells only. Recent studies have shown that tumor cell-fibroblasts cocultures can be beneficial to improve tumor models [30,31,32,33,34]; nevertheless, the application of such a coculture to test Mg-based biomaterials for antitumor activity is to our knowledge entirely new. Specifically, for a model studying the interaction of bone-derived cells with Mg, Burmester et al. suggested Saos-2 cells to be more appropriate than, e.g., MG63 or U2OS [42,43], based on their study in a monoculture system. Furthermore, Saos-2 is the only cell line of the above-mentioned that is able to mineralize [44,45]. Based on this, we chose Saos-eGFP, which originated from Saos-2 osteosarcoma cells, for our osteosarcoma-fibroblast coculture.

Furthermore, fluorescently labeled cells allowed not only the visualization and distinction of both cell types in the coculture directly on the opaque material but also made this coculture a potent monitoring system. As shown in the microscopic images of Figure 1, the cells were generally viable on all materials. Nevertheless, there were substantial differences between the relative cell numbers on degrading (Mg, Mg–6Ag) and non-degrading (Ti–6Al–4V) material, as shown in Figure 1C–E. On Mg and Mg–6Ag, relative healthy fibroblast numbers showed a cell density-independent tendency towards an increase within the observation time (days 1–7). In contrast to this, relative tumor cell numbers increased on the non-degrading Ti-based material. These effects were material-specific as both cells exhibited rather similar proliferation rates. Compared to the findings in the coculture, tumor cell numbers in the monoculture were decreasing on Mg and Mg–6Ag. This indicated relevant crosstalk between tumor cells and surrounding cells in vivo (in this case, fibroblasts) that could not occur in monoculture. Therefore, investigating materials for their anticancer activity should not only be done in monocultures since the results may not be relevant.

Degradable materials, such as Mg and Mg–6Ag, favored the growth of healthy cells over the malignant cell proliferation, indicating the general suitability of these materials for anticancer approaches. Degradability and accompanying surface effects might be one reason for the differences in relative cell numbers on Mg-based materials and non-degrading controls. Future studies should clarify whether this was due to increased tumor cell cytotoxicity or inhibited proliferation. Furthermore, recent publications have also shown favorable effects for healthy cells of the tumor microenvironment [46,47] and unfavorable effects on osteosarcoma cells [43]. However, these findings have been obtained only in monocultures and majorly using Mg extracts and salt solutions, so these results should be interpreted with caution regarding our results.

Moreover, we compared two different cell densities of the established coculture, namely, a sparse (10,000 cells in total) and a dense (50,000 cells in total) system. For the sparse model, relative tumor cell numbers were significantly lower at day 3 (Mg, Mg–6Ag) and day 7 (Mg) compared to the dense model. Possible explanations included: (I) A different degradation rate, pH, and osmolality of the Mg substrates, (II) A different Mg and Ag release or a different impact of these elements on the cells, (III) A different cell proliferation behavior due to contact inhibition.

It is known that cells can alter Mg degradation, which, in turn, can affect the number of cells surviving on the material surface. However, detailed cell-material interaction remains ambiguous, and cells influence Mg material degradation that appears to be highly cell-type and substrate-dependent. Based on the, respectively, studied monoculture systems, the literature indicates that the degradation may be inhibited [36,37], stimulated [38,39,40], or not affected by cells [48]. Our approach of using such a coculture was new, and, therefore, comparisons must be interpreted with caution. Figure 2A,B shows that the degradation rate of the material was not significantly affected by the different cell numbers in the two coculture models. Moreover, Figure 2C,D proves that the initial different cell numbers in the sparse and dense model remained significantly different after seeding. This confirmed the cell-density independence of the degradation of the studied coculture system. The degradation of Mg-based materials also increases the pH and osmolality of the surrounding [17,41,49,50,51]. Figure 3 shows that neither the pH nor the osmolality was significantly different between the sparse and dense coculture model. Based on the comparison of degrading and non-degrading material, the degradation might have an influence on the relative cell numbers of the coculture. But as shown by the MDR, pH, and osmolality, the differences between sparse and dense coculture seemed not to be majorly influenced/caused by cell-density-dependent altered degradation rates.

Another possibility for the different relative cell numbers between sparse and dense coculture models might be a different amount of released ions during the degradation of Mg-based materials. Mg and Ag may act extracellularly or intracellularly with the cells. Therefore, the concentrations of Mg and Ag in the supernatant of degrading Mg and Mg–6Ag were measured by AAS and ICP-MS. We quantified Mg concentrations between 1–6 mM in the supernatant, which was in the expected range supported by other studies [52,53,54,55,56,57]. In general, no significant correlation between ion release and cell ratios could be stated. For Mg–6Ag, the release of Mg and Ag tended to be higher in the control and dense model compared to the sparse model. Since the MDR of the control, dense, and sparse model did not significantly differ, a degradation influence could be excluded. Though, we could measure a significant difference in a few samples. Lower concentrations might be explained by ions bound to degradation products on the material surface. Since the quantification of Mg and Ag was performed using the supernatant of the immersion test, accumulated silver on the material or insoluble degradation products, such as Mg(OH)_2_ or MgCO_3_, could not be taken into account for AAS and ICP-MS [17,49]. Alternatively, the different concentrations between sparse and dense models might be explained by an increased uptake of Mg and Ag by cells. In contrast to healthy cells, tumor cells have a certain avidity for extracellular Mg, which results in increased Mg accumulations in the tumor cells [58]. Mg is involved in the regulation of various enzymes involved in glycolysis or metabolism of nucleic acids and proteins [59,60,61], underlining its proliferation-stimulating effect in tumor cells. This was in contradiction to our findings, in which relative tumor cell numbers decreased on Mg-based material. A possible explanation might be the antagonism of Mg to calcium (Ca). Sun and colleagues reported an increased extracellular serum Ca/Mg ratio in prostate cancer patients. They could see that the increased Ca/Mg ratio in vitro resulted in a massive Ca influx, followed by increased tumor cell proliferation [62]. Consequently, shifting the ratio towards the magnesium would inhibit Ca influx and tumor cell proliferation. These findings were also supported by the work from Pereira et al. The authors observed an inhibited chemically triggered Ca influx through the plasma membrane of MCF-7 cells in the presence of high extracellular Mg levels [63]. Normally, Ca influxes the cells following absorption to mitochondria and the endoplasmic reticulum. Ca-dependent processes like cell proliferation result in depletion of Ca in the endoplasmic reticulum, which again triggers Ca influx, a process called store-operated calcium entry [64,65]. Increased external Mg levels have been reported to inhibit this process [66,67]. Consequently, this would reduce cell proliferation in theory, supporting our findings for the relative cell numbers on Mg and Mg–6Ag from Figure 1D,E.

Furthermore, cells of the tumor stroma, such as fibroblasts, are affected by Mg. Yue et al. showed that Mg supplementation decreased the production of matrix metalloproteinases (MMP) in fibroblasts [28]. These MMPs are important for remodeling the extracellular matrix and subsequent migration and invasion of adjacent tumor cells. Ag is influencing tumor cells mainly via the interaction with proteins and the DNA [68]. This leads to proliferation inhibition [69], induces oxidative stress [70], or even apoptosis [68,71]. Nonetheless, the question arises whether the released Ag concentration in our study is sufficient to induce tumor cell death while preserving healthy cell viability. Literature indicates a large concentration span of IC_50_ (half maximal inhibitory concentration) of Ag sources for different tumor types. The IC_50_ of AgNO_3_ has been reported at 6.75 µM for H-ras transformed 5RP7 (rat embryonic fibroblasts) [72] and between 0.19 and 0.37 mM for Hela cells [73]. For Ag-ions and colloidal Ag, other authors have reported an IC_50_ of 0.31 mM and 32.45 µM, respectively, for breast cancer cells [19,74]. Our results showed that the concentration of released Ag (0.7 nM) was not comparable to the IC_50_ values reported in the other publications. From our data, it was unclear up to what extent the released Mg and Ag affected both cell types. This requires the visualization and quantification of intracellular free or bond Mg and Ag, as well as the investigation of the aforementioned relevant pathways in future studies. The aim of our study was to specifically harm the tumor cells with minimal damage to the healthy cells. This might not only be achieved by Ag but as a result of synergistic effects of Ag and Mg and hydrogen gas (H_2_) evolution. H_2_ has been already studied to induce apoptosis in lung cancer cells [75]. Furthermore, Nan et al. reported H_2_ ability to scavenge free radicals in tumor cells. Interestingly, they generated H_2_ from degrading Mg, suggesting Mg as a material with anti-bone cancer properties [76].

Aside from this, significantly increased relative tumor cell numbers in the dense model compared to the sparse model can be explained by contact inhibition. For healthy cells, cell proliferation progresses until close cell-cell contacts suppress further cell proliferation. Cells undergoing malignant transformation lose this suppression and are no longer limited to monolayers but grow vigorously in multilayers [77,78,79]. Therefore, a dense cell layer, like in the dense model, inhibits the proliferation of healthy cells, without affecting malignant cells. Consequently, the relative tumor cell number increases. 

Another aspect that could be discussed here is the mechanical properties of the used biomaterials. Indeed, cell behavior is greatly influenced by the surface stiffness, as well as the topography of a material [80]. Here, the material topographies did not change significantly over time. However, compared to titanium, the magnesium materials have mechanical properties closer to the ones of bone. For example, the tensile strength of Ti–6Al–4V and cortical bone vary between 895 and 930 MPa and 35 and 283 Mpa [81], respectively, while the one of Mg and Mg–6Ag is about 108.3 ± 3.1 Mpa and 215.9 ± 11.3 Mpa, respectively [82]. The Young’s moduli, a common measure of material stiffness, of Ti–6Al–4V, Mg materials, and bones are about 110–114 Gpa, 45 ± 1 Gpa, and 5–23 Gpa, respectively. Cancerous tissues have generally different mechanical characteristics (increased rigidity or stiffness) compared to healthy tissue, and these variations have been shown to influence the migration, proliferation, and metastasis of cells [83,84,85]. As such, the mechanical properties of the magnesium materials may also have a negative influence on the progression of the osteosarcoma cells. Further investigations on the mechanical properties of the Mg-degradation layer overtime are currently performed.

Our results indicated the suitability of Mg-based materials as antitumor systems. Regarding the here applied coculture consisting of the tumor and healthy cells, degrading materials seemed to increase relative cell numbers of healthy cells compared to non-degrading materials. Additionally, both cocultures—the sparse and dense system—were suitable mechanistic models to investigate different conditions after implantation of the materials. The sparse model might be applied to simulate the implant environment after a partial bone tumor resection, while the dense model might serve as a system to test unresectable areas.

## 4. Materials and Methods

### 4.1. Cell Culture

Human osteosarcoma cell line Saos-2, constitutively expressing the enhanced green fluorescent protein (eGFP), was provided by the research group of Prof. Tognon (Saos-eGFP). The engineered cell line Saos-eGFP was genetically modified to express constitutively the enhanced green fluorescent protein. Cell line characterization details can be found here [86]. Red fluorescent primary human dermal fibroblasts expressing FP602 (RF fibroblasts) were obtained from Innoprot (Innoprot, Derio, Spain). Both cell types were cultured in Dulbecco’s Modified Eagle Medium GlutaMAX-I (DMEM GlutaMAX-I; Life Technologies, Darmstadt, Germany) supplemented with 10% fetal bovine serum (FBS, Merck KGaA, Darmstadt, Germany) under cell culture conditions (37 °C, 5% CO_2_, 95% relative humidity (rH)).

### 4.2. Material Surface Treatment and Cleaning Procedure

Mg (99.95%) and Mg–6Ag (Mg with 6 weight % Ag) were produced by permanent mold gravity casting (Helmholtz-Zentrum Geesthacht, Geesthacht, Germany) and extruded into rods (10 mm diameter). The rods were processed (9 mm diameter) and cut into disks (1.5 mm thickness; Henschel KG, Munich, Germany). Disks were ground (Saphir 360 from ATM GmbH, Mammelzen, Germany) on both sides using SiC 2500 grid paper (Starcke GmbH & Co.KG, Melle, Germany) at 80 rpm. Afterward, the samples were cleaned ultrasonically (Branson 1210, Branson Ultrasonics, Danbury, USA) for 20 min each in n-hexan, acetone, and 100% ethanol and sterilized in 70% ethanol (all chemicals from Merck KGaA, Darmstadt, Germany). Samples were then immersed in 2 mL DMEM supplemented with 10% FBS, starting the degradation experiments without cells, or preincubated in the medium for 24 h prior to experiments, including cells. Ti–6Al–4V (control) samples were cut from a round bar (F.W. Hempel Legierungsmetall GmbH and Co. KG, Oberhausen, Germany; 10 mm diameter × 2 mm height). Samples were polished, ultrasonically cleaned in 2% Hellmanex II solution (Hellma Materials GmbH, Jena, Germany), chloroform, and 100% ethanol (both chemicals from Merck KGaA, Darmstadt, Germany) (20 min each), and sterilized, as described above.

### 4.3. Osteosarcoma-Fibroblast Coculture on Mg-Based Materials

For coculture experiments, Saos-eGFP and RF fibroblasts were dissociated using 0.05% trypsin-EDTA (Life Technologies GmbH, Darmstadt, Germany). The cells were then counted using a CASY Cell Counter (Roche Diagnostics GmbH, Mannheim, Germany), and a cell suspension with a 1:1 ratio of Saos-eGFP and RF fibroblasts was prepared. Monocultures of 10,000 Saos-eGFP or RF fibroblasts on material served as additional controls. A volume of 40 µL containing 10,000 or 50,000 cells in total was directly seeded on the material surface and allowed to adhere for 20 min under cell culture conditions. Afterward, media (2 mL of DMEM supplemented with 10% FBS) were added to each well and changed every 2–3 days. Corresponding materials without cells (Mg, Mg–6Ag), non-degrading material (Ti–6Al–4V), cells on tissue culture plate (cell control), and medium without material and cells (medium control) were selected as controls.

Microscopic images of the whole material surfaces were taken one, three, and seven days after cell seeding using an upright fluorescence microscope (Eclipse Ni-E; Nikon GmbH, Dusseldorf, Germany) and analyzed using ImageJ (Rasband, W.S., ImageJ, U.S. National Institutes of Health, Bethesda, Maryland, USA, https://imagej.nih.gov/ij/, 1997–2018). Cell numbers of both cell types were counted by analyzing particles and calculated as the ratio of the total cell number. The proportion of material covered with cells was determined as the quotient of the area covered by cells and the total surface area of the material.

### 4.4. Determination of Mean Degradation Rate, pH, and Osmolality

The mean degradation rates of the Mg-based materials (with or without cells) were measured by mass loss. Therefore, between the cleaning step with 100% ethanol and sterilization in 70% ethanol, the samples were dried and weighted using a micro-scale (Scaletec, Scaltec Instruments GmbH, Göttingen, Germany) to measure the initial weight. The samples were then immersed in a 24-well plate (Greiner Bio-One International GmbH, Kremsmünster, Austria) with 2 mL DMEM supplemented with 10% FBS under cell culture conditions for seven days. Afterward, samples were rinsed in ultrapure H_2_O and ethanol and dried. To remove the residual degradation layer, the samples were immersed in chromic acid (180 g/L in distilled water, VWR International, Darmstadt, Germany) for 10 min, upended, and immersed for another 10 min. Samples were again rinsed in ultrapure H_2_O and ethanol to remove residual chromic acid and dried. Afterward, the mass was measured again using a micro-scale and the mean degradation rate (MDR), as the average of the degradation rates (DR, *n* = 6) was calculated according to the following equation [87]:

(1) $$DR=\frac{K\times W}{A\times T\times D}$$

*K* is a constant (*K* = 8.76 × 10^4^ for degradation rate in mm/a), *W* is the mass loss in g, *A* is the area in cm^2^, *T* is the immersion time in h, and *D* is the density in g/cm^3^.

Supernatants were taken on days 1, 3, and 7 after cell seeding for analysis of pH and osmolality, and the fresh medium was added on these time points. The pH was measured using a pH meter with an ion-sensitive field-effect transistor (ISFET) pH sensor (Sentron SI600, Sentron Europe BV, Roden, The Netherlands). The osmolality was measured using the freezing point osmometer ‘Osmomat Auto’ (Gonotec GmbH, Berlin, Germany).

### 4.5. Interferometry

Surface topography of the initial and degraded material was conducted via white light interferometry using a non-contact interferometer (contour GT-K, Bruker Corporation, Billerica, MA, USA). Samples were either in the initial state or after degradation for seven days (sparse and dense model) after removing the degradation layer using chromic acid. The raw data was analyzed using the software “Vision64” (v. 4.51, Bruker Corporation, Billerica, MA, USA). First, resulting images of the material surface were corrected regarding their form (cylindrical) and tilted with an F-operator as well as cropped with a circle mask. The 3D Analysis Tool “S Parameters-Height” determined the average roughness (Sa), the maximum peak height (Sp), and the maximum valley depth (Sv) to compare respective samples with the two different seeding densities.

### 4.6. Quantification of Mg and Ag Contents in Degradation Supernatants

Due to possible emission spectrum interferences between Ag and Mg, element releases (Ag and Mg) during degradation were quantified either via AAS (for Mg samples) or ICP-MS (for Mg–6Ag samples). Prior to AAS analysis, the supernatants were acidified with 1 % (w/v) nitric acid (HNO_3_, Merck KGaA, Darmstadt, Germany) and kept at 4 °C to avoid precipitation. Then, samples were diluted 1:250 in 1% HNO_3_ in ultrapure H_2_O, and total Mg concentration was detected using a flame AAS (Agilent 240 AA, Agilent Technologies, Waldbronn, Germany) at a wavelength of 285.2 nm (emission spectrum of Mg). A calibration curve ranging from 0.05 to 1 mg/L Mg was applied for quantification. ICP-MS was applied to quantify the concentration of Mg and Ag in the supernatant after Mg–6Ag degradation. To avoid contaminations, digiTUBEs (S-prep GmbH, Überlingen, Germany) were flushed twice with ultrapure water and 1% (w/v) HNO_3_ in ultrapure water. Supernatants acidified in 1% (w/v) HNO_3_ were diluted 1:1000–2000 in 1% (w/v) HNO_3_ in ultrapure water to a final volume of 30 mL in digiTUBES. The concentrations of Mg and Ag were determined by an inductively-coupled plasma-mass spectrometer (Agilent 7900 ICP-MS, Agilent Technologies, Waldbronn, Germany) with an ESI PFA microflow nebulizer (Elemental Scientific, Omaha, NE, USA).

### 4.7. Statistics

The data were obtained from three independent experiments with three samples and are shown as the arithmetic mean ± standard deviation (SD), if not stated otherwise. Statistical analysis (Prism 6, GraphPad Software, La Jolla, CA, USA) was conducted in two ways, either comparing the mean of respective samples of the dense and sparse coculture model using an unpaired non-parametric t-test (Mann–Whitney test) or comparing both models with a no-cell control using non-parametric one-way analysis of variances (ANOVA) (Kruskal–Wallis test) with Dunn’s multiple comparison test.

## 5. Conclusions

In this study, we used a coculture model of the tumor microenvironment (osteosarcoma-fibroblast) that allowed the direct observation of cell population evolution, especially on opaque material, thanks to cell-specific fluorescence.

Depending on the degradation rate, Mg and Mg–6Ag decreased relative tumor cell numbers in contrast to non-degrading Ti-based material. This effect was assumed to be degradation-dependent since the proliferation rates of the used healthy and tumor cells were found to be comparable. From our preliminary data, we concluded that the used Mg-based materials rather exerted cytostatic than cytotoxic effects on the tumor cells, therefore lowering the relative number of tumor cells. Increasing the degradation rate of the material accompanied by increasing surface effects (pH, osmolality, H_2_ release) might result in rather cytotoxic effects. Comparing a sparse (10,000 cells in total) and dense (50,000 cells in total) variant of the coculture model, the proportion of fibroblasts tended to be higher in the sparse coculture model. This indicated better effects of Mg-based material surrounded by only a few tumor cells, as it could be expected in vivo directly after tumor resection. Although the cell densities were significantly different, the degradation influence on the coculture did not seem to be different between both coculture models, indicating cell-density independence. Further studies should focus on the crosstalk of fibroblasts and tumor cells in this coculture dependent on Mg material degradation. It is also conceivable to test other cells of the TME or extend the present coculture with additional relevant cell types (macrophages, endothelial cells). Additionally, to mimic better the tumor microenvironment, a more complex in vitro system will be developed to also monitor the effect of Mg and its alloys on angiogenesis (with endothelial cells) and inflammatory environment (with macrophage-immune cells).

We concluded that the sparse coculture model, showing more resolute effects with Mg, is a suitable mechanistic system to study possible antitumor effects of Mg-based materials, which will be part of our future work.

## Abbreviations

AAS	Atomic absorption spectroscopy
ICP	Inductively-coupled plasma
MDR	Mean degradation rate
MS	Mass spectrometry
OS	Osteosarcoma
TME	Tumor microenvironment
